# Adoptive T-cell therapy to treat liver cancer: Is the liver microenvironment key?

**DOI:** 10.18632/oncotarget.1179

**Published:** 2013-07-15

**Authors:** Gerald Willimsky, Ulrike Protzer, Percy Knolle, Mathias Heikenwalder

**Affiliations:** Institute of Immunology, Charité Campus Buch, Berlin, Germany, Max Delbrück Center for Molecular Medicine, Berlin, Germany; Institute of Virology, Technische Universität München / Helmholtz Zentrum München, München, Germany; Institutes of Molecular Medicine and Experimental Immunology, University of Bonn, Bonn, Germany, Institute of Molecular Immunology, Technische Universität München, München, Germany; Institute of Virology, Technische Universität München / Helmholtz Zentrum München, München, Germany

Hepatocellular carcinoma (HCC) reflects the 5^th^ most common cause for cancer related deaths worldwide with approximately 800.000 deceases per year and is the third most frequent cause for death worldwide [1]. In African or Asian countries HCC has become the most common cause for cancer-related death, mainly as a consequence of viral infections with Hepatitis B and C-viruses (HBV; HCV). Notably, in Europe and the USA HCC is on the rise, reflecting the fastest increasing cancer type in recent years in the USA. Prospectively this trend will worsen in the USA and Europe since e.g. HCV had already superseded HIV as a cause of death. Moreover, in industrialized countries not only HBV- or HCV-infections surmount this development but more and more high fat diet induced non-alcoholic steatohepatitis (NASH) and non-alcoholic fatty liver disease (NAFLD) lead to liver cancer [1]. Given the effort that has been invested in the past year to understand and treat HCC - either induced by viruses, high fat diet or chronic alcohol consumption - clinical success has been relatively small. Up to now the most effective intervention to prolong life-span of HCC patients has been liver transplantation for those with a single, small nodule – which is the exception. Chemo- and radiotherapy do not lead to an amelioration of the patient's status but rather to tumor cell resistance in patients with HCC. Moreover, the aberrant liver function of HCC patients rather reduces the efficacy of the chemotherapy than promoting it. Various novel drugs have been investigated, and the pantyrosine kinase inhibitor *Sorafenib* [1] has become the standard of care for HCC therapy. Except transplantation, these regimens, however, are rather palliative than curative [1]. Thus, apart from the demand to broaden HCC surveillance programs to reduce overall disease specific mortality for people at risk, an efficient therapy to treat HCC is urgently needed. In contrast to the antigenic heterogeneity of high fat diet or alcohol induced HCC, that express tumor-associated (self) antigens or individually mutated antigens, tumor cells of virus-induced HCC equally express - and may be addicted to - viral proteins, that are recognized by the immune system. Nevertheless, virus-induced HCC often escapes immune-surveillance. In a related virus-induced HCC mouse model we have shown that tumor-specific T cells are functionally activated by the virus infection but that the developing HCC escapes destruction since T cells can only poorly infiltrate HCC and the few remaining, infiltrating T cells are rendered dysfunctional in the liver [2]. Therefore, immunotherapy for treating virus-induced HCC, in particular adoptively transferred T-cells targeted against viral oncogenes acting as shared tumor-specific antigens, could represent a very promising and successful approach.

For adoptive T cell therapy (ATT) two strategies to graft new specificities onto patients T cells are currently pursued and have already shown feasibility in preclinical trials and the clinic: (First) Genetic engineering of T cells with chimeric antigen receptors [3,4] or (Second) with cloned T cell receptors (TCRs; [5]) which recognize tissue/tumor-specific or tumor-associated antigens. Whereas the first is useful to target surface antigens independent of the HLA molecule, the second allows also recognition of antigen cross-presented by the tumor stroma. This has recently been shown to be of utmost importance for preventing tumor recurrence, especially if the targeted antigenic peptides do not have enough affinity for the restricting HLA molecule [6]. Apart from the fact that both types of redirected T cells target foreign viral antigens that are not expressed on non-infected normal tissue, it is advantageous for TCR-redirected T cells that most HBV and HCV viral epitopes, e.g. in the context of the HLA-A2 allele, have a predicted IC_50_ value of high affinity (below 100 nM). Recurrence after appropriate ATT is thus unlikely when tumor growth is dependent on the expression of the respective virus-derived proteins. To enhance the efficacy of ATT and to circumvent negative impact of the tumor microenvironment onto T cells, the transfer of redirected T cells can be accompanied with antibodies e.g. blocking immune regulatory PD-1 or Lag3 pathways. Additionally, the local expansion of adoptively transferred T-cells has to be increased to achieve higher T cell numbers. We have recently investigated this in detail and have found that changing the microenvironment in the liver by activation of toll-like receptors induced the transient existence of intrahepatic myeloid aggregates (iMATEs) which support T cell expansion e.g. enabling control of chronic hepatic viral infection after vaccination with DNA [7]. Thus, these novel applications that allow expansion of T-cells at the site of action could become more efficient to fight liver cancer and preserve the efficacy of adoptively transferred T-cells.

Importantly, it has recently been shown that inflammation, as often accompanied with HCC in patients with chronic HBV or HCV infection, dynamically fine-tunes antigen sensitivity of CD8^+^ T cells that subsequently gain enhanced effector potential to eliminate virus antigen positive cells [8]. However, how chronic inflammation and fibrosis or even acute modulation of the hepatic microenvironment will finally influence the efficacy of adoptively transferred CD8^+^ T cells remains largely unknown and thus has to be investigated thoroughly in future - first in relevant primary mouse models and then translated into the clinic. Accordingly, there are several pivotal questions open in the field of ATT and its possible use for the treatment of liver cancer:

- How to enable efficient anti-hepatoma activity of adoptively transferred T-cells at or within the tumor site?
- How to assure tumor antigen specificity of adoptively transferred T cells in HCC?
- How to control the effect of the liver tumor microenvironment (e.g. inflammation; fibrosis) on T cell expansion, T cell mediated tumor cell killing and survival.

In conclusion, ATT is a highly auspicious strategy to treat HCC. Still, the most efficient modality for ATT for the various HCC subtypes has to be determined, before this approach might become successful in the clinic.
